# Efﬁcacy of orally administered montmorillonite in myoglobinuric acute renal failure model in male rats

**DOI:** 10.22038/IJBMS.2023.67985.14866

**Published:** 2023

**Authors:** Seyed Ali Hoseini azad, Mohammad Moshiri, Ali Roohbakhsh, Abolfazl Shakeri, Ashkan Fatemi Shandiz, Leila Etemad

**Affiliations:** 1 School of Pharmacy, Mashhad University of Medical Sciences, Mashhad, Iran; 2 Medical Toxicology Research Center, School of Medicine, Mashhad University of Medical Sciences, Mashhad, Iran; 3 Department of Clinical Toxicology, Imam Reza Hospital, Mashhad University of Medical Sciences, Mashhad, Iran; 4 Department of Pharmacodynamics and Toxicology, School of Pharmacy, Mashhad University of Medical Sciences, Mashhad, Iran; 5 Pharmaceutical Research Center, Pharmaceutical Technology Institute, Mashhad University of Medical Sciences, Mashhad, Iran; 6 Department of Pharmacognosy, School of Pharmacy, Mashhad University of Medical Sciences, Mashhad, Iran; 7 School of Pharmacy, Mashhad University of Medical Sciences, Mashhad, Iran; 8 International UNESCO Center for Health-Related Basic Sciences and Human Nutrition, Faculty of Medicine, Mashhad University of Medical Sciences, Mashhad, Iran

**Keywords:** Acute renal failure, Bentonite, Clay, Kidney, Montmorillonite, Rhabdomyolysis

## Abstract

**Objective(s):**

Acute kidney injury can be associated with serious consequences and therefore early treatment is critical to decreasing mortality and morbidity rate. We evaluated the effect of montmorillonite, the clay with strong cation exchange capacity, on the AKI model in rats.

**Materials and Methods:**

Glycerol (50% solution, 10 ml/kg) was injected in the rat hind limbs to induce AKI. 24 hr after induction of acute kidney injury, the rats received oral doses of montmorillonite (0.5 g/kg or 1 g/kg), or sodium polystyrene sulfonate (1 g/kg) for three consecutive days.

**Results:**

Glycine induced acute kidney injury in rats with high levels of urea (336.60± 28.19 mg/dl), creatinine (4.10± 0.21 mg/dl), potassium (6.15 ± 0.28 mEq/L), and calcium (11.52 ± 0.19 mg/dl). Both doses of montmorillonite (0.5 and 1 g/kg) improved the serum urea (222.66± 10.02 and 170.20±8.06, *P*<0.05), creatinine (1.86±0.1, 2.05± 0.11, *P*<0.05), potassium (4.68 ± 0.4, 4.73 ± 0.34, *P*<0.001) and calcium (11.15 ± 0.17, 10.75 ± 0.25, *P*<0.01) levels. Treatment with montmorillonite especially at a high dose reduced the kidney pathological findings including, tubular necrosis, amorphous protein aggregation, and cell shedding into the distal and proximal tubule lumen. However, administration of SPS could not significantly decrease the severity of damages.

**Conclusion:**

According to the results of this study, as well as the physicochemical properties of montmorillonite, such as high ion exchange capacity and low side effects, montmorillonite can be a low-cost and effective treatment option to reduce and improve the complications of acute kidney injury. However, the efficacy of this compound in human and clinical studies needs to be investigated.

## Introduction

Acute kidney injury (AKI), previously known as acute renal failure (ARF), results in elevated serum level of creatinine, reduced urine volume, or both (1, 2). AKI can lead to waste product accumulation and electrolyte disorders or even death (3). The rapid deterioration in renal function is associated with worse clinical outcomes and increased risk of long-term chronic kidney disease that can progress to end-stage renal disease, a longer length of stay in the ICU (Intensive Care Unit), and high mortality (4, 5). 

Increased levels of blood urea nitrogen (BUN) and creatinine resulting from the decline in glomerular filtration rate (GFR) is the hallmark of AKI(4). Tubular and glomerular injury are also features of the pathophysiology of AKI (6). Epidemiological evidence has shown that either mild or reversible AKI can be associated with serious consequences and therefore early diagnosis and treatment are critical to decreasing both mortality and morbidity rate (7). The mortality rate in patients with AKI is as high as 20% which is likely to exceed 50% in ICU patients (8, 9). The treatment for AKI is supportive including avoidance of nephrotoxic medications, hypertension therapy, judicious fluid administration, advanced hemodynamic monitoring, and renal replacement therapy (RRT) (10-12). The spectrum of AKI ranges from minor dysfunction to serious complications that require dialysis (6)*. *

According to the type of AKI (pre-renal, renal (intrinsic), and post-renal failure), several factors can be involved. Rhabdomyolysis is recognized as a leading cause of acute renal failure (ARF) and can occur following severe exercise, severe traumatic crush injury, heat stroke, myopathy, septicemia, drug abuse, and alcoholic intoxication (13, 14). AKI is found in about 13–50% of patients suffering from rhabdomyolysis (13). Rhabdomyolysis is a life-threatening condition in which muscle breakdown and cell disruption lead to the release of creatine phosphokinase (CK), lactate dehydrogenase (LDH), and myoglobin into the interstitial space and plasma (15).

Montmorillonite (MMT), a multifunctional clay mineral and a major active element of bentonite, belongs to the smectite family of clays. The strong fluid absorption and cation exchange capacity make it suitable for use in cosmetics and medicine production, the petroleum industry, wastewater treatment, and removal of organic pollutants, gaseous impurities, heavy metals, and other contaminants (16, 17). It has been reported that the habit of eating soil was employed to cure abdominal pain, dysentery, and food infections (18). Unique characteristics such as a high speciﬁc surface area, strong absorption ability, and high binding affinity to heavy metal ions have allowed MMT to be widely used in biomedical research and therapy(19-21). Strong interactions between some toxins, drugs, and MMT can result in low dissolution, concentration, and absorption rate of the substances in the GI tract (gastrointestinal tract). As a result, MMT is applied as an adsorbent of harmful substances in medicine (21-23).

MMT can efficiently absorb creatinine and accelerate its excretion from the intestine in an acute hypercreatininaemia mouse model (24). Hyperkalemia is also a common complication of acute kidney injury and may be treated by oral MMT administration (25). Hypokalemia was indicated as a main adverse effect induced by MMT and other clay mineral ingestion, due to potassium binding (26-28). Thus, theoretically, this mineral can be a suitable candidate for intestinal dialysis in acute renal failure and reduction of the patient’s need for hemodialysis. We evaluated the validity of this hypothesis. 

## Materials and Methods

***Animals and drugs***

In this experiment, adult male Wistar rats weighing 180–220 g were provided by the Laboratory Animal Unit of Mashhad University of Medical Sciences (MUMS). They were housed in cages under a 12 hr/12 hr light-dark cycle, at room temperature (22–25 °C) with free access to food and water. The Animal Care Committee of Mashhad University of Medical Sciences, Mashhad, Iran, approved the experiments. 

MMT and Glycerol were purchased from Merck Co. (Germany) and Sodium Polystyrene Sulfonate (SPS: Kayexalate) from MODAVA Pharmaceutical Co. (Iran).

***Experimental protocols***

In order to induce AKI, the animals were deprived of water for 12 hr and then received an intramuscular injection of glycerol 50% solution at a dose of 10 ml/kg body weight in their hind limbs(14). The animals were randomly divided into seven groups (n=6 for each group). Four groups were given intramuscular injections of 50% glycerol on the first day of the experiment and distilled water (DW), MMT (0.5 or 1 g/kg), or SPS (1 g/kg, positive control) for the next three days through oral administration. The other three groups only received DW (distilled water) (Control), MM, or SPS.

***Evaluation of blood biochemical parameters***

For evaluation of creatinine, urea, CPK enzyme, calcium, magnesium, potassium, and phosphorus levels, the blood samples were obtained through heart puncture on the fifth day after glycerol injection. The animals were anesthetized by intraperitoneal (IP) injection of ketamine/xylazine combination. Blood samples were collected in non-heparinized tubes and centrifuged at 3500 rpm for 15 min to obtain serum. The serum levels of biochemical parameters were measured by a standard autoanalyzer.

***Survival rates and time estimation***

The rates of mortality and survival were monitored throughout the experiment. The personal observation was conducted every six hours, four times a day. The percentage of survival rate was calculated based on the number of live animals until the end of the experiment in each group in comparison with the control group. The average time of survival was also calculated in the same way.

***Body weight and kidney hypertrophy index***

The body weight of animals was measured on the first day, before the AKI induction, and last day of the experiment. The kidney hypertrophy index was also recorded on the last day. The data were expressed as body weight changes and kidney-to-body weight (KW/BW) ratio. 

***Histological analysis***

Formalin-fixed right kidneys were embedded in paraffin and cut into five μm thickness sections. All slides were stained with hematoxylin and eosin. A blind pathologist reviewed the kind and severity of the lesions. The pathological findings were reported semi-quantitatively as a score ranging from one to three plus (severe = +++, moderate = ++, mild = +, and normal histology = -), equal to 20-25%, 10-20%, 5-10 %, and 0%, respectively (29).

***Statistical analysis***

The results have been presented as mean ± SEM and the evaluation has been done by one-way ANOVA, *post hoc* Tukey’s test using GraphPad Instat software. Survival rates between groups were compared with Fisher’s exact test. Chi-squared test was used for comparing the survival rates of animals. A *P*-value less than 0.05 (< 0.05) was set as statistically significant. 

## Results

***Survival rate and time***

Intramuscular injection of glycerol significantly reduced the survival rate and time compared with the control group (*P*<0.001). Although administration of MMT at doses of 0.5 mg/kg and SPS could not change the survival rate, MMT increased the survival time (*P*<0.05) in comparison with the glycerol group. MMT at the dose of 1 g/kg significantly increased the survival rate and time (*P*<0.001). No significant difference was observed in either parameter in groups that received MMT or SPS alone compared with the control group (Table 1). 

***Blood biochemical parameters***

*Serum urea level*

**Figure 1:**
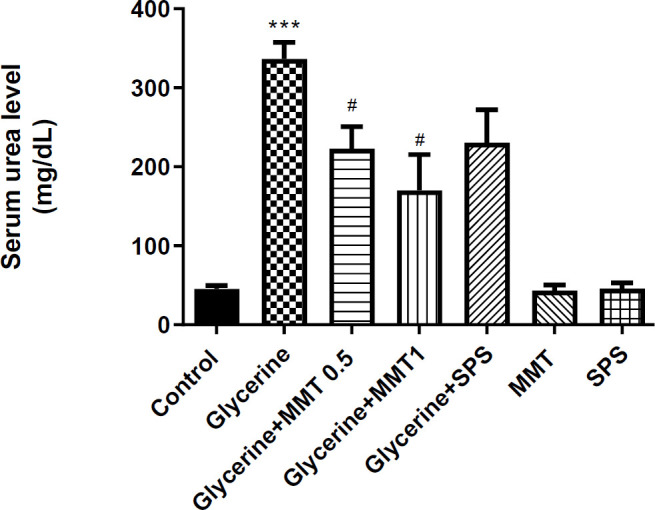
Evaluation of serum urea level in a model of AKI. Animals received intramuscular injections of 50% glycerol on the first day of the experiment and DW, MMT (0.5 or 1 g/kg), or SPS (1 g/kg) for the next three days through oral administration. The other three groups only received DW (Control), MM (1 g/kg), or SPS. For statistical comparison, one-way ANOVA and Tukey–Kramer test were used. Data are expressed as mean±SE. *** *P*<0.001 compared with the control group, # *P*<0.05 compared with the glycerol group

AKI: Acute kidney injury; DW: Distilled water; MMT: Montmorillonite; SPS: Sodium polystyrene sulfonate

Glycerol injection increased the serum urea level compared with the control group (*P*<0.001). Oral administration of MMT at different doses led to a decrease in the serum urea level compared with the glycerol group (*P*<0.05). However, SPS could not induce significant changes in urea levels (Figure 1).

*Serum creatinine level*

**Figure 2:**
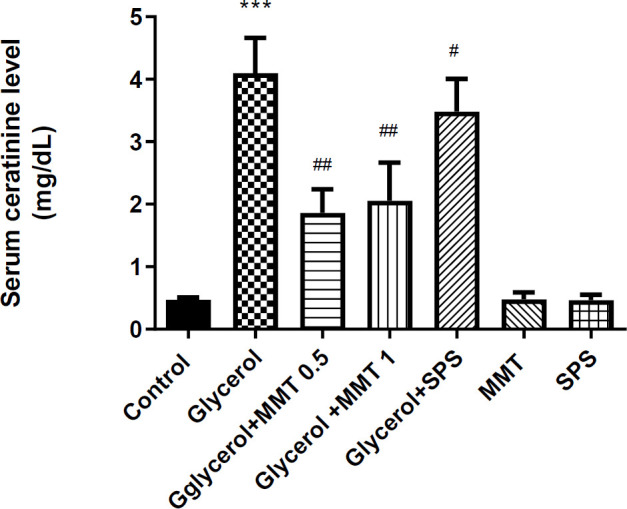
Evaluation of serum creatinine level in a model of AKI. Different groups received glycerol plus DW, MMT (0.5 or 1 g/kg), MMT (1 g/kg), or SPS (1 g/kg). Animals received intramuscular injections of 50% glycerol on the first day of the experiment and DW, MMT, or SPS for the next three days through oral administration. The other three groups only received DW (Control), MM, or SPS. For statistical comparison, one-way ANOVA and Tukey–Kramer test were used. Data are expressed as mean ± SD. *** *P*<0.001 compared with the control group, # *P*<0.05 and ##*P*<0.01 compared with the glycerol group

AKI: Acute kidney injury; DW: Distilled water; MMT: Montmorillonite; SPS: Sodium polystyrene sulfonate

Treatment with MMT at both doses (*P*<0.01) and SPS (*P*<0.05) significantly prevented the glycerol-induced increased level of creatinine. MMT and SPS administration alone did not induce any changes in serum creatinine levels (Figure 2).

*Serum creatine phosphokinase (CPK) level*

**Figure 3:**
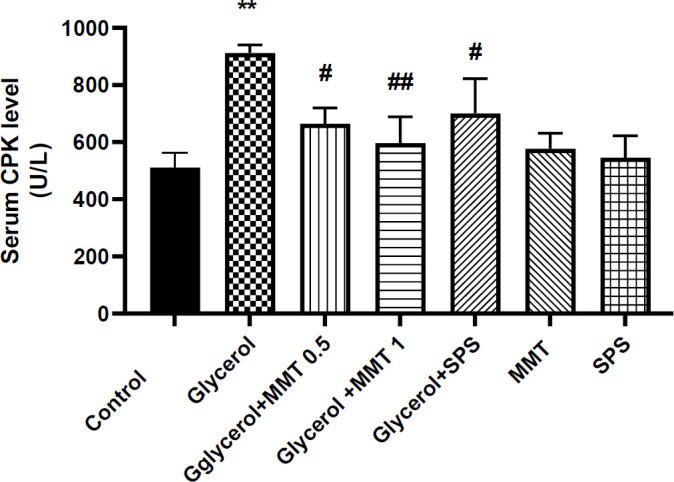
Evaluation of serum CPK level in a model of AKI. Different groups received glycerol plus DW, MMT (0.5 or 1 g/kg), MMT (1 g/kg), or SPS (1 g/kg). Animals received intramuscular injections of 50% glycerol on the first day of the experiment and DW, MMT, or SPS for the next three days through oral administration. The other three groups only received DW (Control), MM, or SPS. For statistical comparison, one-way ANOVA and Tukey–Kramer test were used. Data are expressed as mean±SD. ** *P*<0.01 compared with the control group, # *P*<0.05 and ##*P*<0.01 compared with the glycerol group

CPK: creatine phosphokinase; AKI*: *Acute kidney injury; DW: Distilled water; MMT: Montmorillonite; SPS: Sodium polystyrene sulfonate

Intramuscular injection of glycerol increased serum CPK concentration compared with the control group (*P*<0.01). Oral administration of MMT at doses of 0.5 (*P*<0.05) and 1 g/kg (*P*<0.01), as well as SPS (*P*<0.05) induced a significant decrease in serum CPK level in comparison with the glycerol group. There was no significant difference in serum CPK concentration in groups that received MMT or SPS alone (Figure 3).

*Serum calcium, phosphorus, magnesium, sodium, and potassium level*

Glycerol injection in a mouse model of acute kidney injury resulted in increased calcium (Ca) (*P*<0.05), magnesium (Mg) (*P*<0.001), and potassium (K) (*P*<0.001) levels. MMT at different doses and SPS could significantly reduce the serum Ca and K levels in comparison with the glycerol group. None of the absorbents could alleviate the elevated mg level. No significant differences were observed in the level of phosphorous (P) and sodium (Na) between groups (Table 2).

***Body weight changes and kidney hypertrophy index***

**Figure 4:**
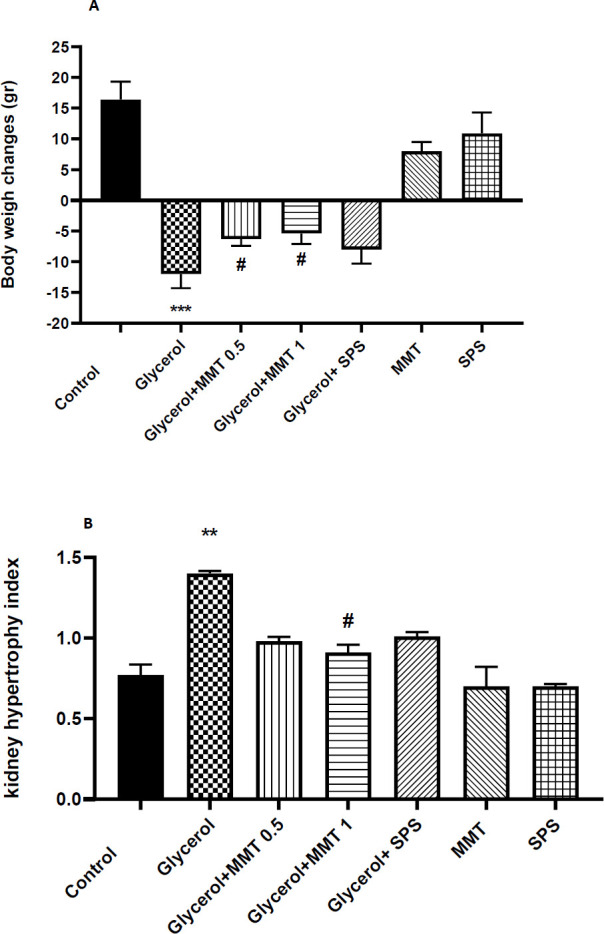
Evaluation of body weight changes (A) and kidney hypertrophy index (B) in a model of AKI. Different groups received glycerol plus DW, MMT (0.5 or 1 g/kg), MMT (1 g/kg), or SPS (1 g/kg). Animals received intramuscular injections of 50% glycerol on the first day of the experiment and DW, MMT, or SPS for the next three days through oral administration. The other three groups only received DW (distilled water) (Control), MM, or SPS. For statistical comparison, one-way ANOVA and Tukey-Kramer test were used. Data are expressed as mean ± SD. ** *P*<0.01, ***P<0.001 compared with the control group, # *P*<0.05 compared with the glycerol group

AKI induced a significant decrease in body weight in comparison with the control group (*P*<0.001) which was reversed by MMT administration at doses of 0.5 and 1 mg/kg (*P*<0.05). SPS could not modify the weight changes (Figure 4A). The ratio of the kidney to rat weight on the last day in the glycerol group showed a significant increase compared with the control group (*P*<001). However, MMT gavage only at a dose of 1 g/kg could attenuate the weight ratio(*P*<0.05) (Figure 4B). 

***Histopathological effects***

**Figure 5:**
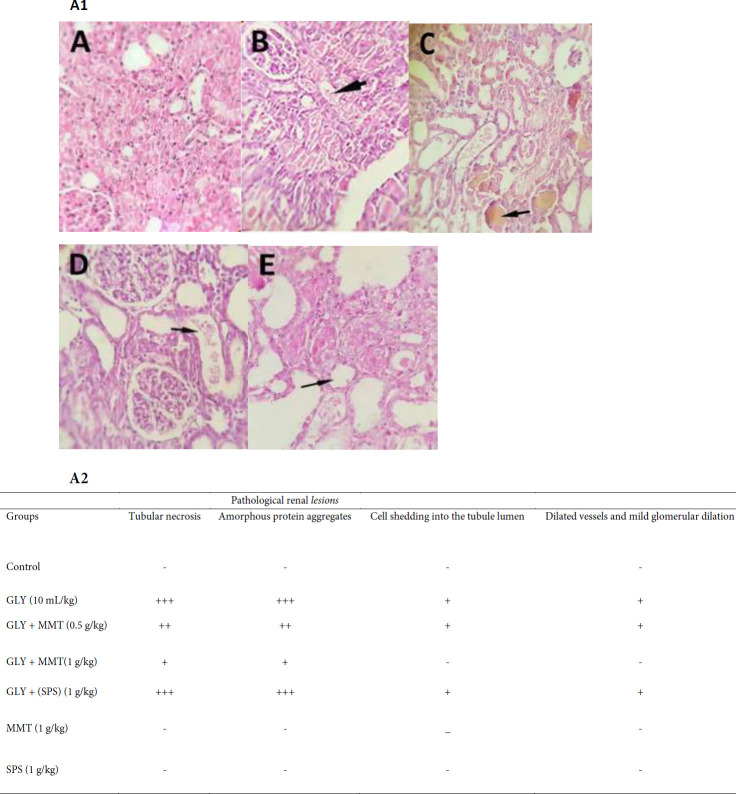
A1) Photomicrographs of hematoxylin and eosin-stained sections of renal tissue of rat. Different groups received glycerol plus DW, MMT (0.5 or 1 g/kg), MMT (1 g/kg), or SPS (1 g/kg). Animals received intramuscular injections of 50% glycerol on the first day of the experiment and DW, MMT, or SPS for the next three days through oral administration. The other three groups only received DW (Control), MM, or SPS. A) Control group with normal tissue structure, B) High tubular necrosis and amorphous protein aggregation in the group-received glycerol, C) Tubular necrosis up to 20% in the group that received glycerol plus MMT 0.5 g/kg, D) Tubular necrosis up to 10% in the that received glycerol plus MMT 1 g/kg (magnification: 10 x 20x), and E) tubular necrosis up to 25% in the group that received glycerol plus SPS. A2) Histopathological damages in the kidney were evaluated and the score was between severe = +++, moderate = ++, mild = +, and normal histology

DW: Distilled water; MMT: Montmorillonite; SPS: Sodium polystyrene sulfonate

Pathological findings demonstrated that glycerol injection could lead to acute kidney damage including, abundant tubular necrosis in cortical tubules, presence of amorphous protein aggregation in tubules, cell shedding into the tubule lumen, relatively dilated vessels, and mild glomerular dilation. Administration of MMT at a dose of 0.5 g/kg attenuated the lesions by reduction of tubular necrosis by about 10–20%, decreasing the deposition of amorphous protein (medium) and relatively dilated vessels. MMT at a dose of 1 g/kg resulted in a marked reduction in kidney injury by decreasing the tubular necrosis to 5–10% and a very low deposition of amorphous protein in renal tubules. However, administration of SPS could only slightly reduce the severity of the damage. The observed lesions were 20–25% of necrosis of cortical tubules, high presence of amorphous protein, and cell shedding into the tubule lumen. No pathological finding was reported in the control and groups that received only MMT or SPS (Figure 5).

**Table 1:** Effect of Montmorillonite on survival rate and time in a rat model of acute kidney injury

Groups	Survival rate (percentage)	Survival time (hours) Mean ±SE
Control	100	120.00±0.48
GLY (10 mL/kg)	50 ***	72.75±4.72 ***
GLY + MMT (0.5 g/kg)	50	102.86±6.85 #
GLY + MMT (1 g/kg)	83.3 ###	116/57±3.42 ###
GLY + (SPS) (1 g/kg)	66.6	96.00±5.8
MMT (1 g/kg)	100	120.10±0.48
SPS (1 g/kg)	100	120±12.48

Evaluation rate and time of rat survival in a model of AKI. Animals received intramuscular injections of 50% glycerol on the first day of the experiment and DW, MMT (0.5 or 1 g/kg), or SPS(1 g/kg) for the next three days through oral administration. The other three groups only received DW (Control), MM (1 g/kg), or SPS. One-way ANOVA and Tukey–Kramer test were used for statistical comparison of the means of survival times. chi-squared test was used for comparing the Survival rates of animals. ***P<0.001 compared with the control group and # *P*<0.05 and ### *P*<0.001 compared with the glycerol group

AKI: Acute kidney injury; DW: Distilled water; MMT: Montmorillonite; SPS: Sodium polystyrene sulfonate

**Table 2.** Effect of Montmorillonite on serum calcium, phosphorus, magnesium, sodium, and potassium levels in a rat model of Acute kidney injury

Groups	Ca (mg/dl)	P (mg/dl)		Mg (mg/dl)	K(mEq/L)	Na(mEq/L)
Control	**0.78** **±** **9.76**	**11.52 ** **±** ** 0.23**		**2.29 ** **±** ** 0.13**	**4.84 ** **±** ** 0.36**	**140.22 ** **±** ** 9.6**
Glycerol	**11.52 ** **± ** **0.19** *	**11.34 ** **±** ** 0.48**		**3.35 ** **±** ** 0.31** ***	**6.15 ** **±** ** 0.28** ***	**139.78 ** **±** ** 8.9**
Glycerol+ MMT 0.5	**11.15 ** **±** ** 0.17** **#**	**10.55 ** **±** ** 0.45**		**3.76 ** **±** ** 0.21**	**4.68 ** **±** ** 0.4** **#** **#** **#**	**140.38 ** **±** ** 10**
Glycerol + MMT 1	**10.75 ** **±** ** 0.25** **#** **#**	**10.86 ** **±** ** 0.87**		**3.52 ** **±** ** 0.59**	**4.73 ** **±** ** 0.34** **###**	**139.14 ** **±** ** 8.8**
Glycerol + SPS	**11.30 ** **±** ** 0.16** **#**	**10.64 ** **±** ** 0.74**		**3.38 ** **±** ** 0.76**	**4.91 ** **±** ** 0.31** **#** **#** **#**	**139.68 ** **±** ** 9.4**
MMT	**9.21 ** **±** ** 0.44**	**10.93 ** **±** ** 0.95**		**2.55 ** **±** ** 0.41**	**4.63 ** **±** ** 0.41**	**139.96 ** **±** ** 6.8**
SPS	**9.92 ** **±** ** 0.45**	**11.07 ** **±** ** 0.39**		**2.13 ** **±** ** 0.26**	**4.53 ** **±** ** 0.38**	**139.82 ** **±** ** 7.2**

Different groups received glycerol plus DW, MMT (0.5 or 1 g/kg), MMT (1 g/kg), or SPS (1 g/kg). Animals received intramuscular injections of 50% glycerol on the first day of the experiment and DW, MMT, or SPS for the next three days through oral administration. The other three groups only received DW (Control), MM, or SPS. For statistical comparison, one-way ANOVA and Tukey–Kramer test were used. Data are expressed as mean ± SD. *P<0.05 and *** *P*<0.001 compared with the control group, # *P*<0.05, ##*P*<0.01, and ###*P*<0.001 compared with the glycerol group

AKI: Acute kidney injury; DW: Distilled water; MMT: Montmorillonite; SPS: Sodium polystyrene sulfonate

AKI*: *Acute kidney injury; DW: Distilled water; MMT: Montmorillonite; SPS: Sodium polystyrene sulfonate

## Discussion

Clay minerals are good adsorbents for organic and inorganic molecules due to their large speciﬁc surface area. MMT is one of the most-used clays that possess high adsorption ability of materials (30, 31). *In vitro* studies have shown that MMT can bind to heavy metals and has the ability to absorb organic compounds and bacteria (32-37). Our previous results revealed that administration of MMT to iron-poisoned and lithium-intoxicated rats reduced the serum mineral levels and thus the symptoms of poisoning (18, 22). The results from another unpublished study also indicated that MMT was able to prevent the absorption of acetaminophen in the intestine similar to “infinite sink” or “intestinal dialysis “method. In this kind of dialysis, an absorbent substance is placed in the intestine and blood entering the intestines acts like a dialysis system (38). Urea, creatinine, and other waste products, which diffuse into the GI (gastrointestinal tract) from the blood, can bind to absorbents like activated charcoal and excrete in the feces, thus the created concentration gradient, in the name of intestinal dialysis, causes continuation of the diffusion (39). In many patients with AKI, dialysis may be needed to help replace kidney function. We suggest that MMT with high absorption capacity might create a new compartment in the intestine and decrease the patient’s need for hemodialysis. 

Outcome and complications of AKI such as retention of nitrogenous waste products and increase in the concentration of creatinine in blood have boosted the mortality rate and decreased quality of life in patients (40). Induction of AKI by glycerol leads to rhabdomyolysis that manifests by increased creatine phosphokinase and is followed by the accumulation of proteins resulting from muscle cell damage such as myoglobin within the renal tubules (amorphous deposits), renal tubular cell necrosis, and renal edema (41). In the present study, MMT could reverse the glycerol-induced increased serum urea, CPK and creatinine level, especially at high doses. BUN levels increase with decreased renal function in acute or chronic renal failure. MMT has been shown to accelerate the release of urea from blood vessels into the intestine and prevents urea absorption in the intestine (42). Urea is easily trapped in MMT spaces. It was stated that about 33.7% of urea can be absorbed into this mineral (43). Cation exchange is one of the proposed mechanisms for the absorption of ammonia into the hydroxyl groups of MMT (44). Therefore, clay minerals can be applied as an effective substance in reducing the effects of nitrogen metabolite. Creatinine is a catabolic product of creatine phosphate in muscles and is considered an important indicator of kidney health. Creatinine is mainly excreted through the kidney and able to distribute in the intestinal tract, especially in renal impairment. It has been stated that the level of creatinine, as well as BUN in serum, were directly correlated with that in the intestinal tract (45, 46). The results of the study showed that MMT could reduce serum creatinine levels in rats through increasing absorption and intestinal secretion (24). Creatinine is able to be released from blood vessels into the intestine and reabsorbed in the intestine (61). Zhang *et al.* showed that MMT reduced the serum creatinine level in rats with hypercreatininemia through creatinine absorption and increased intestinal secretion (62). In the present study, MMT decreased the serum creatinine as well as urea level and improved the renal function in AKI by creating a process similar to intestine dialysis. 

Other manifestations of rhabdomyolysis include electrolyte disturbances. Through the course of the process, serum potassium, calcium, and phosphate levels can increase as myocytes are damaged (47). Hyperkalemia may be caused by two mechanisms, potassium leak from myocytes and decrease in GFR (glomerular filtration rate) secondary to acute renal failure. Rhabdomyolysis at the initial step can decrease the calcium levels due to cell membrane damage and calcium penetration into cells that follow by gradually increasing due to re-equilibration. Similar to other searches (48, 49), the results of the present study showed that glycerol injection induced hyperkalemia, hypercalcemia and hypermagnesemia, while the level of phosphorous did not change. MMT administration could correct the glycerol-induced electrolyte abnormalities except for hypermagnesemia. The present study demonstrates for the first time that MMT can reduce and correct the increased level of calcium levels. Various studies have shown that oral administration of clay can cause hypokalemia (26-28, 50, 51). Amanda *et al*. presented a case of severe hypokalemia in a pediatric patient who received oral and rectal bentonite clay. The child was given a home remedy, containing bentonite and MMT, as a treatment for chronic constipation (52). MMT-induced hypokalemia can be caused by interlinear cation exchange for potassium ions by MMT. However, in our previous published and unpublished data, MMT administration did not change the normal potassium levels (22). It appears that this substance can reduce the serum potassium when the level is higher than normal, but in normal conditions does not cause a severe decrease in potassium levels. In agreement with this evidence, in the present study, the group that received MMT alone did not show a change in potassium levels. It was reported that there is a U-shaped relationship between potassium concentration and mortality in renal insufficiency, depending on the stage of kidney disease and age. It was reported that potassium levels of less than four and more than five mmol/L are associated with high mortality rates (60). Our results showed that the glycerol-received group with high potassium levels, had also a higher mortality rate and it was almost reversed by MMT administration at the dose of 1 g/kg. 

Skeletal muscle damage from rhabdomyolysis can result in the excessive release of myoglobin into the bloodstream and exert a toxic effect on renal tubules (53). Myoglobin easily passes through the pores of the gastrointestinal tract and is secreted in the small intestine (54, 55). As a non-specific adsorbent, MMT clay can rapidly absorb myoglobin as well as many other proteins such as lysozyme, pepsin, etc., and lead to AKI healing process acceleration (56, 57). 

In the present study, the glycerol group showed the highest weight loss and KW/BW ratio. It was reported that glycerol-induced acute renal injury leads to body weight loss and kidney weight gain in rats (42). Our results indicated that body and renal weight changes were alleviated through oral MMT administration.

SPS is a well-known treatment for hyperkalemia resulting from renal failure(58), which can be associated with gastrointestinal problems and intestinal necrosis (59). The mechanism of SPS and MMT in the treatment of hyperkalemia is similar; both of them trap potassium secreted in the intestines. The rate of potassium excretion in normal conditions is about 95% from the kidneys and 5% from the intestines which increases in renal failure diseases (60). The administration of adsorbents such as SPS and MMT not only increases the concentration gradient but also prevents reabsorption of potassium in the colon. Although SPS is a proper adsorbent for the treatment of hyperkalemia in renal failure, its use is limited due to gastrointestinal side effects (61). However, the results of the present study showed that SPS could not increase the survival time of rats. SPS administration was also unable to reduce the urea level and alleviate the weight changes. The pathological findings also confirmed the disability of SPS to attenuate the tissue lesions. However, MMT especially at high doses could almost completely reduce the pathological changes.

## Conclusion

Oral MMT administration may serve as an appropriate therapeutic agent with few side effects in the treatment of AKI. MMT, especially at high doses, is capable to alleviate glycerol-induced AKI via improving the biochemical outcome and pathological abnormality. It is essential to conduct further investigation in order to determine the exact protective effect of MMT on patients suffering from AKI. 

## Authors’ Contributions

LE and MM designed the experiments; SAHA performed experiments and collected data; LE, AFS, MM, AR, and AS analyzed and interpreted results; LE, MM, and AR supervised, directed, and managed the study; SAHA, AFS, and AS prepared the draft manuscript; LE, MM, SAHA, AF, AS, and AR approved the final version to be published.

## Conflicts of Interest

The authors declare no conflicts of interest.
